# 3D nanofiber scaffolds from 2D electrospun membranes boost cell penetration and positive host response for regenerative medicine

**DOI:** 10.1186/s12951-024-02578-2

**Published:** 2024-06-08

**Authors:** Lingfei Xiao, Huifan Liu, Huayi Huang, Shujuan Wu, Longjian Xue, Zhen Geng, Lin Cai, Feifei Yan

**Affiliations:** 1 Department of Spine Surgery and Musculoskeletal Tumor, Zhongnan Hospital of Wuhan University, Wuhan, 430071 China; 2 Department of Anesthesiology, Research Centre of Anesthesiology and Critical Care Medicine, Zhongnan Hospital of Wuhan University, Wuhan, 430071 China; 3 Department of Respiratory and Critical Care Medicine, Renmin Hospital of Wuhan University, Wuhan, 430071 China; 4 The Institute of Technological Science, School of Power and Mechanical Engineering, Wuhan University, Wuhan, 430072 China; 5 Institute of Translational Medicine, Shanghai University, Shanghai, 200444 China; 6 National Center for Translational Medicine (Shanghai) SHU Branch, Shanghai University, Shanghai, 200444 China

**Keywords:** Electrospun nanofiber membranes, Three-dimensional scaffolds, Tissue engineering, Macrophage polarization, Mesh-like

## Abstract

The ideal tissue engineering scaffold should facilitate rapid cell infiltration and provide an optimal immune microenvironment during interactions with the host. Electrospinning can produce two-dimensional (2D) membranes mimicking the extracellular matrix. However, their dense structure hinders cell penetration, and their thin form restricts scaffold utility. In this study, latticed hydrogels were three-dimensional (3D) printed onto electrospun membranes. This technique allowed for layer-by-layer assembly of the membranes into 3D scaffolds, which maintained their resilience impressively under both dry and wet conditions. We assessed the cellular and host responses of these 3D nanofiber scaffolds by comparing random membranes and mesh-like membranes with three different mesh sizes (250, 500, and 750 μm). It was found that scaffolds with a mesh size of 500 μm were superior for M2 macrophage phenotype polarization, vascularization, and matrix deposition. Furthermore, it was confirmed by subsequent experiments such as RNA sequencing that the mesh-like topology may promote polarization to the M2 phenotype by affecting the PI3K/AKT pathway. In conclusion, our work offers a novel method for transforming 2D nanofiber membranes into 3D scaffolds. This method boasts flexibility, allowing for the use of varied electrospun membranes and hydrogels in terms of structure and composition. It has vast potential in tissue repair and regeneration.

**Supplementary Information:**

The online version contains supplementary material available at 10.1186/s12951-024-02578-2.

## Introduction

Tissue engineering offers a promising solution to tissue repair and regeneration [1, 2]. The design and construction of tissue engineering scaffolds is essentially the art of arranging specific spaces such as channels and pores within the scaffold. Such porous scaffolds offer a 3D supportive environment conducive to cell growth [3, 4]. The scaffold’s porosity, pore dimensions, and surface attributes optimize nutrient and oxygen delivery, guiding cellular behavior and thereby directing tissue restoration [5–7]. 

Electrospinning offers capabilities for fabricating materials at a much finer scale compared to other techniques such as freeze-drying, sacrificial templating, 3D printing, and melt electrowriting [8]. It is a versatile method for generating ultrafine fibers ranging from nanometer to micrometer scales. The resulting membranes support cell adhesion, nutrient transfer, and new tissue formation [9, 10]. The topographical cues on the electrospinning membrane can modulate individual cell morphology and overall cell patterning, subsequently determining cell fate [11]. For instance, electrospinning fibers with parallel alignment have been found to promote the polarization of macrophages toward an M2 phenotype [12, 13]. Furthermore, a mesh-like electrospinning membrane encouraged the secretion of anti-inflammatory and pro-angiogenic cytokines by adipose-derived mesenchymal stem cells [14]. In contrast, a latticed electrospinning membrane could upregulate the HIF-1 signaling pathway, which promotes vascularization and enhances bone regeneration [15]. However, the density of the membranes hinders cell infiltration, and their thinness, existing as a flexible two-dimensional surface, restricts their use as scaffolds [16]. 

The conversion of two-dimensional nanofiber membranes into 3D nanofiber scaffolds broadens the applications of electrospinning [17, 18]. Notably, such scaffolds can be used to address defects in three-dimensional spaces, specifically in bones and cartilage, which harness the benefits of nanofibers creating a conducive environment for cells mirroring their native in vivo [19]. A variety of fabrication techniques exist for these 3D scaffolds, including multilayering electrospinning, sacrificial agent electrospinning, wet electrospinning, ultrasound-enhanced electrospinning, and several post-processing methods like short fiber assembly, gas foaming, ultrasonication, and electrospraying [17–20]. Among these, the short fiber assembly method stands out. In this method, the electrospinning membrane is fragmented into short fibers, which are then reconstructed into a sponge-like or 3D-printed scaffold. Such scaffolds have interconnected pores fortified with these short fibers, optimizing them for cell adhesion, nutrient transport, and immunomodulation [21–23]. Gas foaming is another notable technique. It involves stretching the dense electrospinning mat using air bubbles. This action sporadically connects the layers to form a three-dimensional scaffold. Impressively, this technique preserves the original aligned topological characteristics, which play crucial roles in directing cell migration and enhancing tissue growth and bone regeneration [24, 25]. However, many of these methods fall short in manifesting detailed topological features on the scaffold’s surface, like precise porosity and patterning. The layer-by-layer stacking technique fills this gap. It merges layers created via other methods with those of electrospun ones. For instance, merging electrospinning layers with 3D-printed ones could steer immune polarization towards M2, which was conducive to vascularization and bone regeneration [26]. Further, combining the melt electrowriting mesh with electrospinning layers yielded a lightweight scaffold, which minimally triggered foreign body reactions while ensuring excellent biocompatibility [27]. However, in the layer-by-layer stacking method, the electrospun layers in previous studies only constituted a minor portion of the scaffold, failing to harness the full potential of nanofibers in mimicking the 3D extracellular matrix. Moreover, studies focusing on the influence of membrane topography on the efficacy of tissue engineering scaffolds remain sparse.

**Fig. 1:**
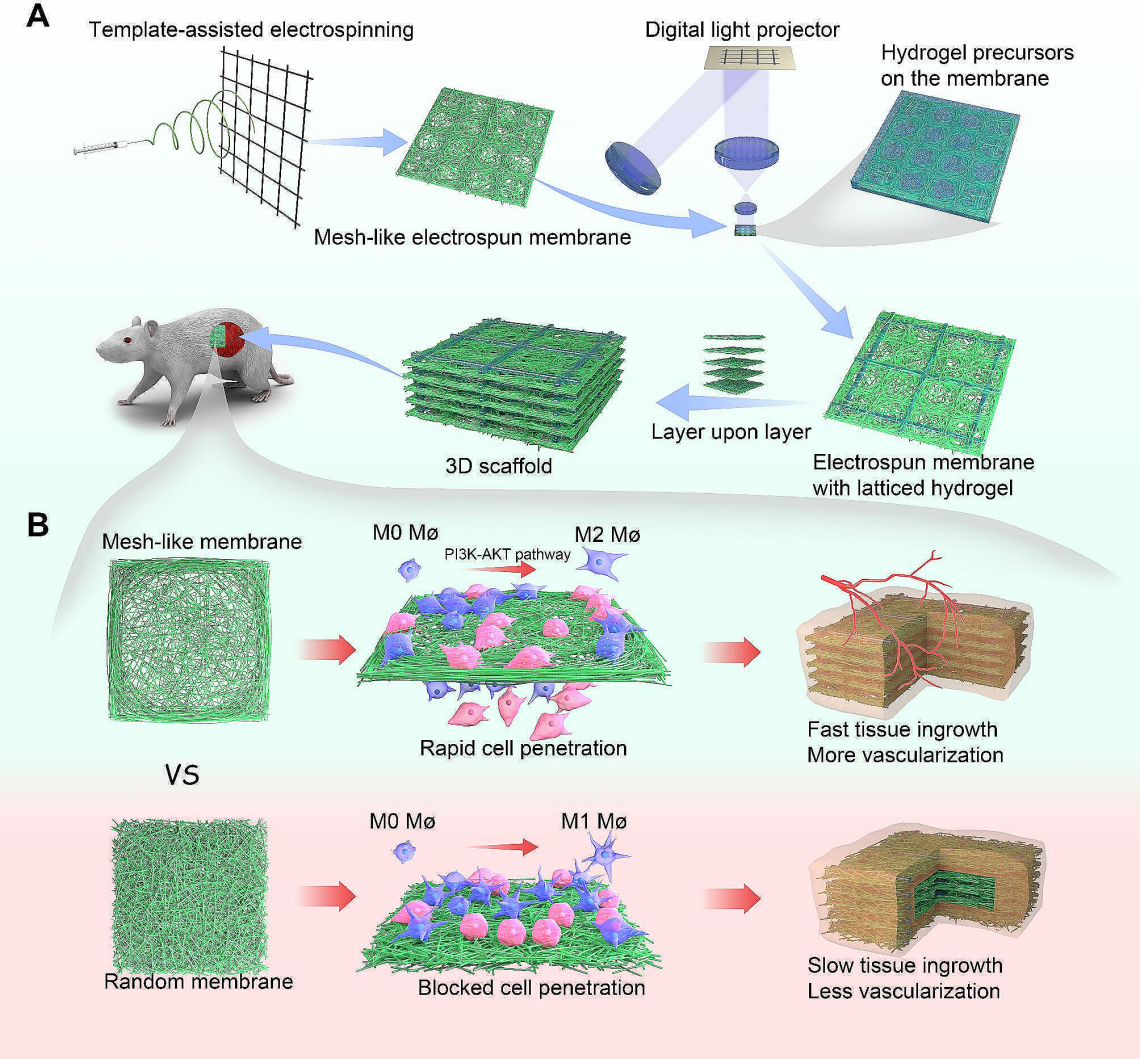
Schematic overview of the research. **(A)** Production of a mesh-like porous nanofiber membrane using a template-assisted electrospinning technique. This was followed by the fabrication of a latticed hydrogel layer on the nanofiber membrane using 3D printing technology, which was then sequentially assembled to obtain a three-dimensional nanofiber scaffold. **(B) **Three-dimensional scaffolds composed of mesh membranes of appropriate size proved advantageous for rapid cell infiltration and macrophage polarization to the M2 phenotype when compared to those composed of unordered membranes. Ultimately, this facilitates faster tissue integration and increased vascularization

Here, we combined template-assisted electrospinning with DLP (Digital Light Processing)-based 3D printing technology. A small amount of latticed hydrogels were printed on electrospun membranes, enabling the membranes to be assembled layer by layer into a three-dimensional scaffold, ensuring inter-layer spaces for cell growth. In addition, the pattern of the monolayer membrane can be tailored based on the received template (Fig. 1A). Considering the effects of membrane pores and topological morphology on cell permeation and immune response, we prepared three-dimensional scaffolds consisting of random membranes and mesh-like nanofiber membranes with three different mesh sizes (250, 500, and 750 μm). Through in vitro cellular experiments and subcutaneous embedding experiments, we have found the most suitable 3D scaffolds for rapid tissue ingrowth and good immune response, which will provide a reference for future applications in tissue repair (Fig. 1B).

## Materials and methods

### Materials

PCL (Mw ≈ 80,000) and silkworm cocoon were purchased from Macklin Co., Ltd. (Shanghai, China). Sodium bicarbonate, lithium bromide, hexafluoro isopropanol (1,1,1,3,3,3-hexafluoro-2-propanol, HFIP), and lithium phenyl-2,4,6-trimethylbenzoylphosphinate (LAP) were obtained from Sigma-Aldrich (Shanghai, China). RPMI-1640, α-MEM, Penicillin-streptomycin, Fetal bovine serum (FBS), Phosphate buffer saline (PBS), and Trypsin were purchased from HyClone Laboratories Inc. (UT, USA). Gelatin methacryloyl (GelMA) (Degree of substitution: 90%) was purchased from Cure Gel Co., Ltd. (Wenzhou, China). For the cell culture experiment, fetal bovine serum (FBS), Dulbecco’s modified Eagle’s medium (DMEM), alpha-modified Eagle’s medium (α-MEM), phosphate-buffered saline (PBS), trypsin-EDTA, and penicillin/streptomycin (P/S) were purchased from HyClone Laboratories Inc. (UT, USA). The Cell Counting Kit-8 (CCK-8) was obtained from Dojindo Laboratories, Kumamoto, Japan. The live/dead cell staining kit was supplied by BestBio Biotechnologies (Shanghai, China). Triton X-100 (Sigma–Aldrich), 4′,6-diamidino-2-phenylindole (DAPI, Solarbio), and Cy3-labeled phalloidin (Solarbio) were used for cell staining. All the antibodies used in this study were purchased from Abclone Technologies Co., LTD. (Wuhan, China).

### Extraction of silk fibroin

Silk cocoons were sectioned into approximately 25 mm^2^ segments and submerged in boiling 0.2 M sodium bicarbonate solution, stirred constantly to enhance the interaction between the silk and the alkaline medium. After degumming, the silk underwent rigorous rinsing with ultrapure water before drying for subsequent applications. A concoction, comprising 20 g of lithium bromide, 25 g of water, and 6 g of degummed silk, was uniformly blended and then placed in a 55 °C water bath for 4 h until it clarified. Dialysis was then performed on this mixture using a bag with a molecular weight cut-off of 8,000, against ultrapure water at 4 °C. This four-day procedure involved bi-daily water changes to expel the lithium bromide. Following dialysis, the solution underwent centrifugation at 4,500 rpm to discard insoluble matter. The resulting solution was frozen, lyophilized, and conserved at -20 °C for subsequent applications.

### Preparation of nanofiber membrane

A solvent was formulated by dissolving 0.8 g of PCL and 0.2 g of Silk Fibroin (SF) in 10 ml of hexafluoroisopropanol. Then it was mixed up and down with a rotary mixer for 12 h to achieve a transparent solution. This solvent was loaded into a 5 ml plastic syringe for electrospinning. An electrospinning device, sourced from Yongkang Le Industry Company, China, was utilized. Preset parameters included a steady ambient temperature and humidity, a 22G needle, 1 ml/h spinning rate, 16 kV voltage, 20 cm spinning distance, and a collector of either tin foil or stainless steel mesh with assorted pore diameters (250, 500, 750 microns). The random or mesh-like electrospun membrane underwent immersion in methanol for 15 min, facilitating the transformation of the α-helix in silk fibroin to a β-sheet configuration.

### Preparation of three-dimensional nanofiber scaffold

The random or mesh-like electrospun membrane was placed in DLP (Digital Light Processing)-based 3D printer (nanoArch S130, BMF Precision Tech Inc.). The membrane was coated with a photocured hydrogel precursor solution (0.25% LAP in 5%GelMa aqueous solution, a commonly used photocured hydrogel formulation). The printer was then used to produce a lattice of UV light projection areas (lattice strips: 0.2 mm wide, lattice sizes: 1.5 × 1.5 mm squares). The UV wavelength in the printer was 405 nm, and it was irradiated for 5s at a time, for a total of 10 times. Then a latticed hydrogel layer (approximately 250 microns thick) was crosslinked on the electrospun nanofibers. The uncrosslinked precursor solution was then thoroughly washed away. We carefully manipulated two tweezers, stacking layers of electrospun membrane with latticed hydrogel on top of each other to create a 3D scaffold. The 3D scaffold was then further crosslinked using a UV flashlight for the second time. The latticed hydrogel dominated the thickness of scaffold, so it taked about 20 layers to get a 5 mm thick scaffold.

### Characterization

The scaffolds were freeze-dried and then subjected to various characterization tests. The chemical scaffolds and crystalline structures of the materials were analyzed using a Fourier transform infrared (FTIR) spectrometer (Nicolet 5700, Perkin–Elmer) and an X-ray diffractometer (Rigaku D/max 2400 diffractometer, Tokyo, Japan). The surface of the gold-sputtered materials was observed under a scanning electron microscope (MIRA3, LMH, TESCAN), and the diameter of the fibers was measured and documented using the FIJI software. The water contact angle (WCA) was assessed by employing a video contact angle tester (FM40MK2, Kruss). For mechanical tests, a universal testing machine (Bose ElectroForce 3200, Bose CROP.) was employed where the dry samples were cut into dumbbell shapes with a width of 5 mm for tensile testing and square shapes with 5 mm sides for compression tests, with each set of samples being tested five times.

### In vitro biocompatibility

Before cell cultivation, the scaffolds were soaked in alcohol for 4 h, and then were washed with PBS three times for 15 min each time. The extracts of the scaffolds were obtained according to the international standard ISO 10993-12: 2012. The sterilized scaffold was immersed in 0.1 g/mL (w/v) α-MEM culture medium and incubated at 37℃ for 3 days. Following this, FBS (10%, v/v) was added to obtain scaffold extracts. The scaffold’s biocompatibility was subsequently assessed with Bone Mesenchymal Stem Cells (BMSCs). Specifically, BMSCs were introduced into 96-well plates at 5.0 × 10^3^ cells/well. After the cells adhered, the medium was replaced with the extract. On days 2 and 4, the optical density at 450 nm was determined after adding CCK-8 solution (Beyotime, China). Meanwhile, some wells were stained with calcein-AM/PI to mark live/dead cells, visualized under a fluorescence microscope. The proportion of viable cells was calculated.

BMSCs were seeded onto sterile scaffolds in 24-well plates at a density of 8.0 × 10^4^ cells/scaffold. On days 2 and 4, live cells were stained with calcein-AM and scrutinized under a confocal microscope to observe adhesion dynamics.

Single-layer electrospun membranes were placed and fixed onto 24-well coverslips. BMSCs were seeded onto these coverslips at a density of 8.0 × 10^4^ cells/scaffold. On days 2 and 4, after removing the membranes from the coverslips, cells on the coverslips were fixed with a 10% formaldehyde solution and stained with 0.1% crystal violet, followed by imaging.

### Quantitative reverse transcriptase polymerase chain reaction (qRT-PCR)

RAW 264.7 cells were cultivated on scaffolds within 24-well plates at 8.0 × 10^4^ cells/scaffold for a 3-day duration. RNA was subsequently isolated using TRIzol® Reagent (Invitrogen, USA), and its concentration and purity were ascertained via the Thermo Scientific NanoDropTM 1000 UV-Vis Spectrophotometer. cDNA synthesis ensued with the FastQuant RT kit. The qRT-PCR was executed with the SYBR Green supermix (Bio-Rad, USA) and recorded on the CFX96 real-time PCR detection system. Relative gene expression levels for IL-1β, IL-6, IL-4, and IL-10 were standardized against GAPDH, and computed employing the 2^−ΔΔC^_T_ method. Primer sequences can be found in Table S2.

### Flow cytometric analysis

Post a 3-day culture period of RAW 264.7 cells on scaffolds in 24-well plates (8.0 × 10^4^ cells/scaffold), the cells were subjected to trypsinization and resuspension in PBS. Cells underwent a 15–30 min primary antibody incubation on ice, followed by two buffer washes. They were then incubated with a fluorescently labeled secondary antibody and subsequently washed twice more. The cells were resuspended in 1X PBS for flow cytometric analysis. FlowJo (Tree Star) software was employed for data processing, setting a 1% gating for isotype controls to negate non-specific staining and comparing percentages of distinct cell populations displaying marker-positive staining.

### Enzyme-linked immunosorbent assay (ELISA)

After culturing RAW 264.7 cells on scaffolds in 24-well plates for 3 days at a density of 8.0 × 10^4^ cells/scaffold, the concentrations of VEGF, TNF-α, and TGF-β in the supernatants were determined by ELISA kits (Elabscience Biotechnology, Wuhan, China). The procedure involved the initial addition of 40 µL of sample diluent to the ELISA plate wells, followed by 10 µL of the sample. Subsequently, 100 µL of enzyme-labeled reagent was added to each well, and the plate was sealed and incubated at 37℃ for 60 min. Post-incubation, the wells were washed, and 50 µL of color reagent A, followed by 50 µL of color reagent B, were added and gently mixed. This was incubated in the dark at 37℃ for 15 min before the reaction was halted with a stop solution. The absorbance was measured at 450 nm using an ELISA reader, and factor concentrations were calculated from a standard curve.

### Inhibition of PI3K/AKT pathway

RAW 264.7 cells were seeded onto Mesh-500 scaffolds in 24-well plates at a density of 8.0 × 10^4^ cells/scaffold and cultured for 24 h. Then, cells were treated with 10 µM of the PI3K inhibitor LY294002 (MedChem Express, Princeton, USA) for 24 h, followed by cultivation in a regular culture medium for an additional 24 h. The expression of CD206, ARG-1, IL-4, and IL-10 in cells was detected using RT-PCR. Cells on the plate and those on the Mesh-500 scaffold without inhibitor treatment served as controls.

### Western blotting

After 3 days of culturing RAW 264.7 cells on scaffolds in 24-well plates at a density of 8.0 × 10^4^ cells/scaffold, total proteins were collected using a Total Protein Extraction Kit (Beyotime Biotechnology, China), and protein concentration was determined by the Bicinchoninic Acid (BCA) method. Sample proteins underwent SDS-PAGE and membrane transfer. The transferred membranes were blocked and then incubated overnight at 4°C with antibodies against Integrin β1, phosphorylated PI3K(p-PI3K), PI3K, phosphorylated AKT (p-AKT), AKT, MCP-1, and Glyceraldehyde 3-Phosphate Dehydrogenase (GAPDH). After incubation with secondary antibodies for 30 min, the blots were visualized by a Tanon-5200 chemiluminescent imaging system (Tanon, Shanghai, China). Results were reproduced in three independent experiments using different samples.

### Rat subcutaneous implantation

Animal experiments were conducted in accordance with the guidelines for the care and use of experimental animals established by the National Institutes of Health (NIH) and ethically approved by the Animal Care and Use Committee of Zhongnan Hospital, Wuhan University (Approval No. ZN2021194). Nanofiber scaffolds were cut into dimensions of 5 mm x 5 mm x 1 mm and sterilized using ethylene oxide gas, followed by immersion in saline solution for reserve. Male SD rats weighing between 250 and 300 g were utilized for subcutaneous implantation. Briefly, the rats were anesthetized with a constant delivery of 4% isoflurane. An area measuring 8 cm ×4 cm was shaved on the dorsal region of each rat, and the povidone-iodine solution was applied thrice to the exposed skin. A 1 cm incision was made at the center, followed by a slight separation of skin and muscles using a toothless tweezer to create a subcutaneous pocket. The scaffold was carefully placed into the pocket, ensuring a distance of more than 3 cm between adjacent scaffolds. The incision was then sutured. Rats were euthanized with CO_2_ at 1 and 2 weeks post-implantation, after which the implanted materials were harvested for histological staining and transcriptome sequencing analyses.

### Histology and immunohistochemical analysis

Specimens were fixed and underwent dehydration and paraffin infiltration using an automated tissue processor, followed by manual embedding and sectioning in paraffin. Hematoxylin & Eosin (H&E) and Masson’s Trichrome staining were performed on the samples. For immunohistochemical staining, slides were deparaffinized, followed by endogenous peroxidase activity elimination via incubation in 3% H_2_O_2_ at room temperature for 5–10 min. This was followed by rinsing with distilled water and a 5-minute PBS soak. Antigen retrieval was achieved by heating in citrate buffer (10 mM, pH 6.0, 95–100 °C) for 10 min. The slides were then blocked with 5–10% normal goat serum (diluted in PBS) for 10 min at room temperature. Appropriate primary antibodies (e.g., CD68, CD206) were applied and incubated at 37 °C for 1–2 h. The biotin-labeled secondary antibody was applied (diluted in 1% BSA-PBS) and incubated at 37 °C for 10–30 min, followed by horseradish peroxidase-conjugated streptavidin (diluted in PBS) for an additional 10–30 min. Visualization was done using a DAB chromogen. After thorough rinsing with tap water, counterstaining was performed, and the slides were sealed. Following slide scanning, average optical density was analyzed using the IHC TOOLBOX in FIJI software.

### Transcriptome sequencing and data processing

Two weeks post-implantation, samples from both the Random and Mesh-500 scaffolds were collected and preserved in RNA Later™ stabilizing solution (ThermoFisher Scientific, USA). Bulk-RNA-Seq was conducted by Biomarker Co., Ltd. (China). Key terms such as immune response, inflammatory response, and angiogenesis were subjected to KEGG and GO enrichment analyses. Heatmaps of differentially expressed genes were constructed to illustrate the levels of gene expression within distinct scaffolds. Furthermore, key gene expressions (Acta2, CCL2, CXCL1) were verified via RT-PCR.

## Results and discussion

### Preparation and characterization of 3D scaffold

**Fig. 2:**
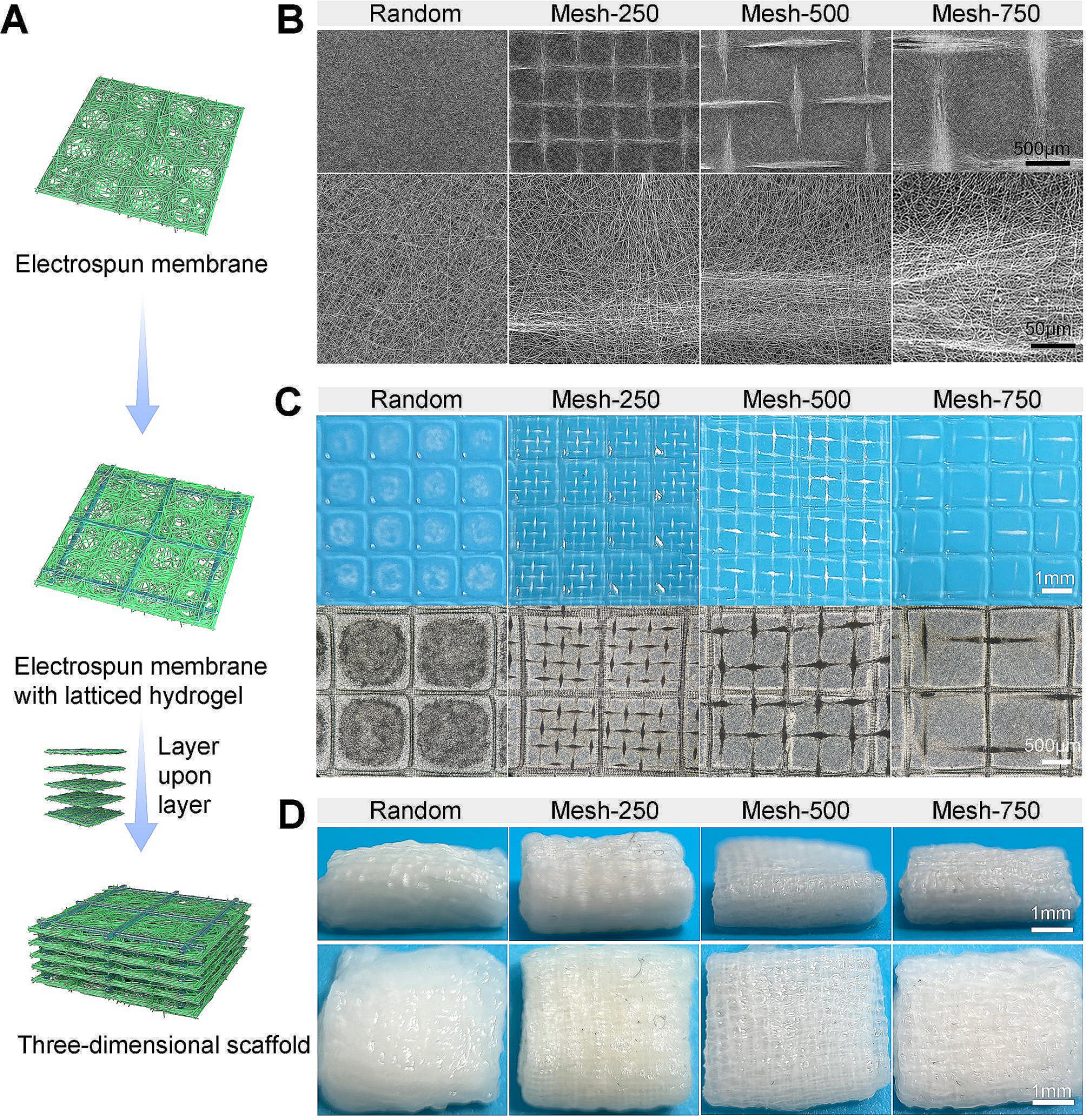
Fabrication and characterization of the three-dimensional nanofiber scaffold. **(A)** Schematic of the fabrication process (with corresponding physical images on the right).** (B)** SEM images of the unordered and three different mesh specifications of the electrospun membranes (Random, Mesh-250, Mesh-500, Mesh-750). The top row displays low magnification, while the bottom row showcases high magnification images. **(C)** Photographs of the electrospinning membrane integrated with a latticed hydrogel layer. **(D)** Lateral and top-down views of the three-dimensional nanofiber scaffold

We proposed a strategy to fabricate a porous 3D nanofiber scaffold: creating a porous nanofiber membrane through template-assisted electrospinning, followed by the development of a latticed hydrogel layer using 3D printing, and finally, layer-by-layer assembly to form a 3D scaffold (Fig. 1A). Tin foil and metal meshes of different sizes (with pore sizes of 250 μm, 500 μm, and 750 μm) were used as receiving templates to produce random and mesh-like membranes (Random, Mesh-250, Mesh-500, Mesh-750). Given the significant impact of membrane thickness on the scaffold’s post-implantation performance, we carefully examined the reception time for a single layer. When the time was too short, the electrospinning membrane became too thin and brittle, affecting the 3D assembly; if too long, the mesh-like morphology became blurred. Ultimately, the receiving duration was set at 40 min. For the region of nanofiber deposition, nanofibers tended to deposit both on the receiving metal wires and the blank areas. The larger the mesh size, the more nanofibers settled on the metal wires and fewer in the blank areas. Those nanofibers deposited on the metal wires aligned approximately parallel to the major axis of the wire, while those in the blank areas exhibited an interlaced overlapping arrangement (Fig. 2A, B). The nanofibers of membranes have a diameter of 650 ± 200 nm (Fig. S1). Subsequently, Gelatin methacryloyl (GelMA) hydrogel was selectively solidified onto the membrane using a 3D printer. This design ensures the separation of adjacent electrospun membranes, offering space for tissue growth. Therefore, the dimensions of the latticed hydrogel were not designed too densely, preserving the effectiveness of the electrospinning membrane. The latticed hydrogel was uniformly distributed on the membrane (Fig. 2C). In the non-photo-cured areas of GelMA, no residual GelMA was found; they were completely washed away with water without damaging the appearance of the membrane. Sequentially, composite layers of the electrospinning membrane and hydrogel were stacked to form a 3D scaffold. It can be seen that the layers are well isolated and loose and porous (Fig. 2D). Then the 3D scaffolds were freeze-dried to facilitate long-term storage at room temperature. The weight of the scaffolds increases to about four times its original weight after water absorption, which may be related to its high porosity (70–85%) (Table S1). In addition, the scaffolds were super hydrophilic and the droplets were absorbed the moment they were dropped on the scaffolds (Fig. S2), which was attributed to the addition of silk fibroin in the electrospinning (Fig. S3). The addition of silk fibroin to the electrospinning polymer has been reported to improve the biocompatibility of the material, modulate the immune response, and accelerate tissue repair [28, 29]. 

### Mechanical properties of the 3D scaffold

**Fig. 3:**
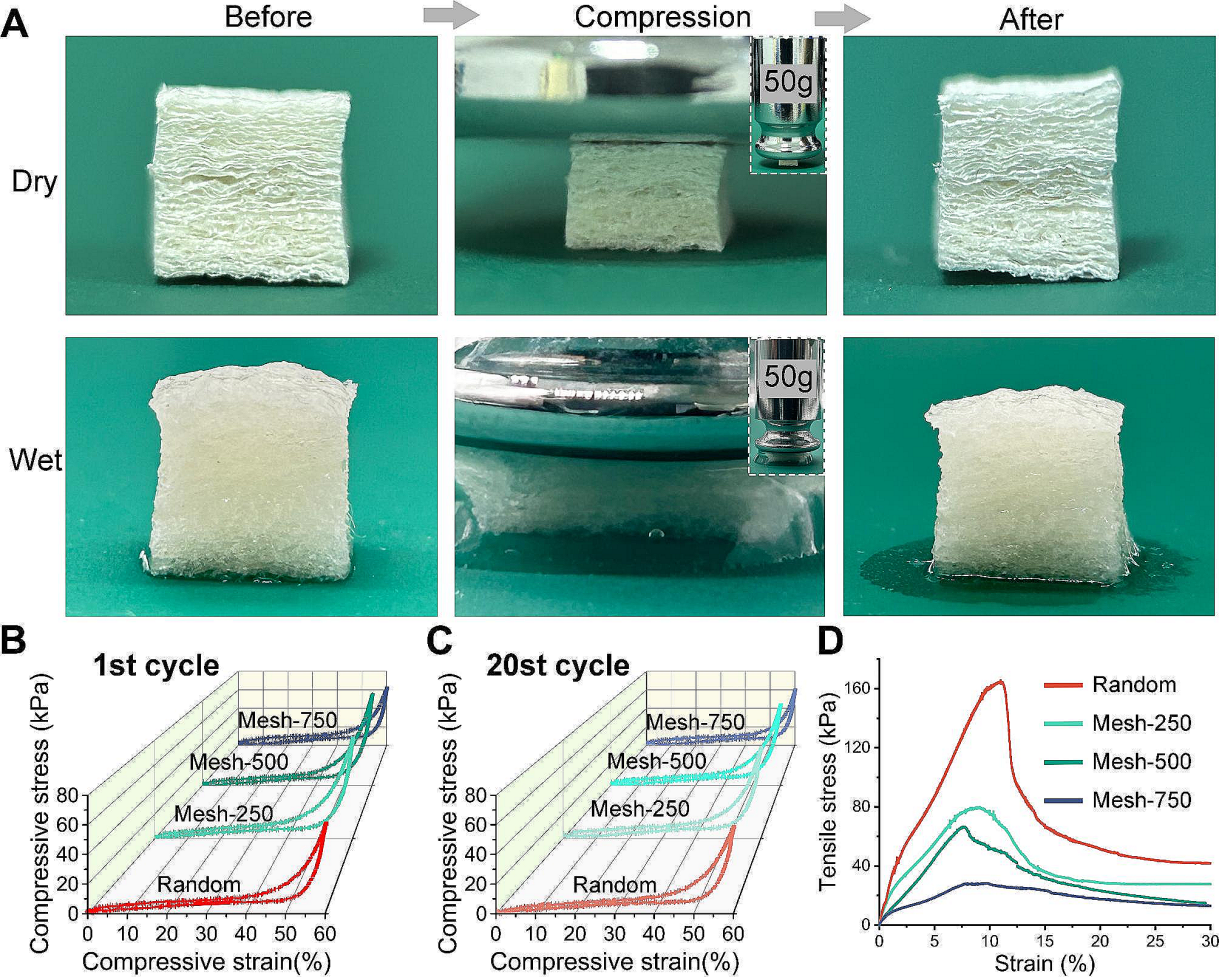
Mechanical properties of the three-dimensional nanofiber scaffold. **(A)** Images illustrating the scaffold’s ability to return to its original form after compression by a 50 g weight, in both dry and wet conditions. **(B)** Cyclic compression tests of scaffolds under a 60% compressive strain (1st cycle). **(C)** Cyclic compression tests of scaffolds under a 60% compressive strain (20th cycle). **(D)** Tensile testing of the scaffolds

The scaffolds were trimmed into cubes of 5 × 5 × 5 mm, showcasing good resilience. Regardless of their dry or wet state, they returned to their original form after being pressed with a 50 g weight (Fig. 3A, Video 1). Mechanical performances of the scaffolds were assessed via cyclic compression and tensile testing. Cyclic compression tests revealed that after 20 compression cycles under a maximum strain of 60%, the stress-strain curves of the scaffolds almost matched the initial cycle (Fig. 3B, C), with recovery rates being Random (96 ± 4%), Mesh-250 (97 ± 3%), Mesh-500 (98 ± 2%), and Mesh-750 (97 ± 2%). This resilience might be attributed to the latticed hydrogel undergoing deformation upon compression, while the electrospun membrane provided support, preventing easy rupture. Tensile tests indicated that the Random scaffold exhibited the best tensile properties, followed by Mesh-250, Mesh-500, and Mesh-750 (Fig. 3D). This could be because as the mesh size increases, the nanofibers are more orderly aligned, but there are imperfections at the metal mesh nodes, resulting in less tight binding of the nanofibers, causing them to break at these defect sites. For tissue engineering scaffolds, good elasticity ensures the scaffold conforms better to tissue defects, achieving better integration with the tissue, and a certain degree of tensile resistance ensures the scaffold won’t shatter in the human body as soft tissues deform.

Various strategies for nanofiber three-dimensionalization have been explored over the years. The methods of short fibers and gas foaming have garnered significant attention [19]. Compared to the short fibers method, our method retains the original appearance of the nanofiber membrane, allows for customized surface structures, and does not require cross-linking, while the mechanical properties of the short fibers method depend on the final cross-linking method. For three-dimensional scaffolds made by the gas expansion method. Its structure was not easy to maintain according to our previous attempts. The scaffold relies on the intermittent contact between the membranes after expansion thus supporting the whole three-dimensional form.

### Effects of materials on cells in vitro

**Fig. 4:**
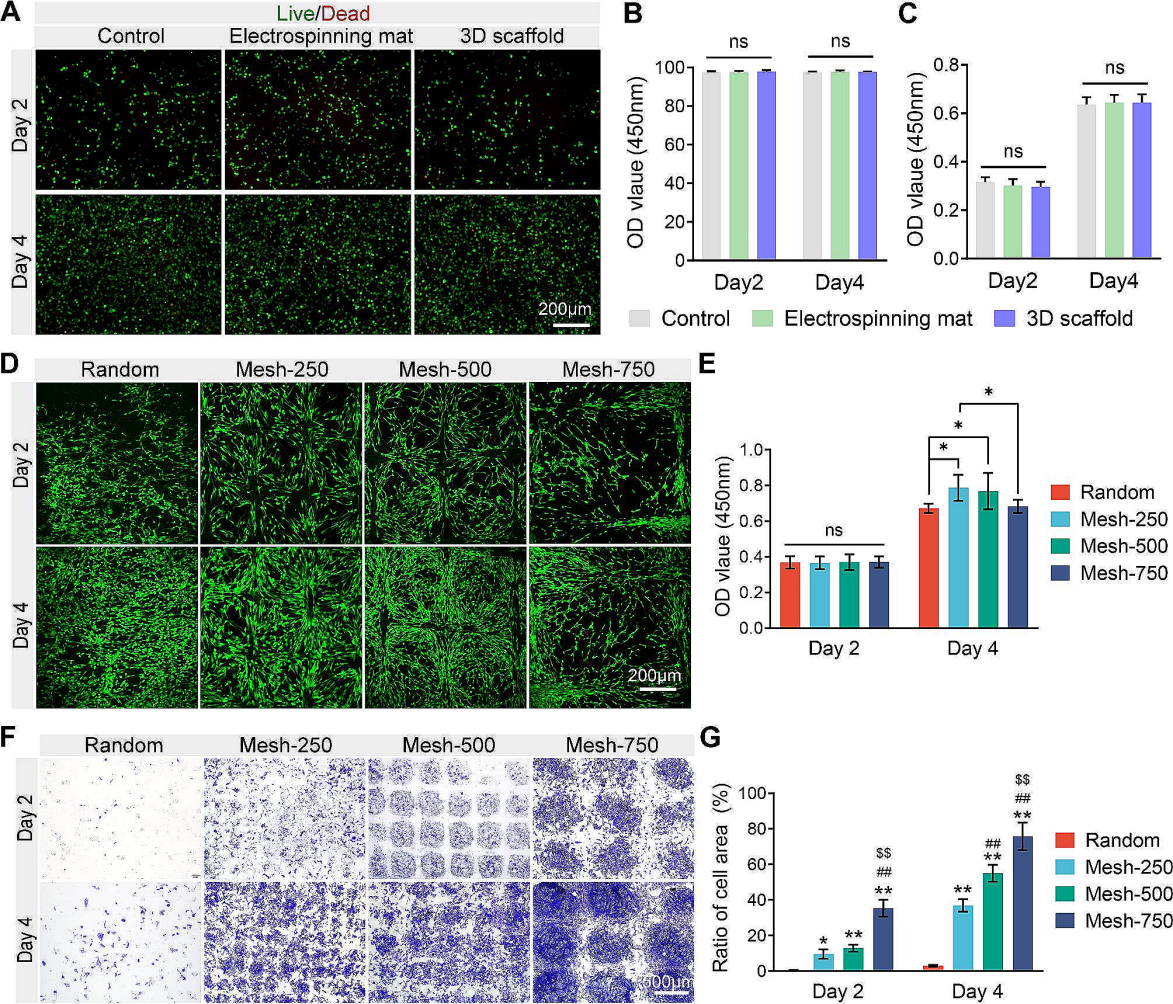
Biocompatibility of the scaffold and its capability to permit cellular migration through the electrospinning membrane. **(A)** Live/dead cell staining of cells cultivated in the scaffold component’s extraction fluid. **(B)** Statistical representation of the live cell percentage from live/dead staining. **(C)** CCK-8 assay of cells cultivated in the scaffold component’s extraction fluid. ns, no significance.** (D)** Confocal microscopy images of live/dead stained BMSC cells post-cultivation on the scaffold. **(E)** CCK-8 assay post BMSC cell cultivation on the scaffold. ns, no significance. *P < 0.05. **(F)** Crystal violet staining images of cells after migration through a single layer of the electrospinning membrane onto a cell round coverslip. **(G)** Percentage representation of the cellular region on the round coverslips. (* and ** represent *P* < 0.05 and *P* < 0.01 when compared with Random. ## represent *P* < 0.01 when compared with Mesh-250. $$ represent *P* < 0.01 when compared with Mesh-500.)

The morphology and structure of scaffolds play pivotal roles in directing cellular responses such as adhesion, migration, and proliferation. We first assessed the cytotoxicity of individual scaffold components. Compared to the control scaffold with standard culture medium, the live/dead cell ratios in extracts of the electrospinning membrane and the 3D scaffold incorporated with hydrogel were both over 95% (Fig. 4A), with no significant difference on day 2 and 4 (Fig. 4B). Similarly, the OD values reflecting cell numbers were consistent on days 2 and 4 (Fig. 4C). This suggests the extracts were non-toxic. Subsequently, upon examining cell-seeded membranes, we found that cell morphology aligned closely with the nanofiber orientation of the substrate. In regions where fibers were orderly aligned, cells adopted an elongated form. And the cells’ distribution often mirrored the scaffold’s mesh pattern (Fig. 4D). CCK-8 experiments revealed no significant difference in cell counts on the various membranes on day 2. By day 4, the Random scaffold had fewer cells compared to Mesh-250 and Mesh-500, while Mesh-750 had fewer than Mesh-250 (Fig. 4E). This suggests that the mesh structure promotes cell proliferation, but overly large pores may reduce cell growth area, leading to a decrease in cell count. Additionally, we placed the membranes on round coverslips, exploring the number of cells that penetrated the electrospun membrane and attached to the coverslips. On day 2, numerous cells had penetrated the Mesh-750 membrane, attaching to the coverslip. Some cells had penetrated Mesh-500 and Mesh-250, but almost none from the Random scaffold. By day 4, a large number of cells were observed on coverslips from the Mesh scaffolds (Fig. 4F), with a sequence of Mesh-750 > Mesh-500 > Mesh-250, while the Random scaffold still exhibited very few cells (Fig. 4G). This indicates that larger pores are more conducive for cell penetration through electrospun membranes, but this difference diminishes over time in mesh-like membranes.

### Tissue integration status of the scaffold in rat subcutaneous implantation

**Fig. 5:**
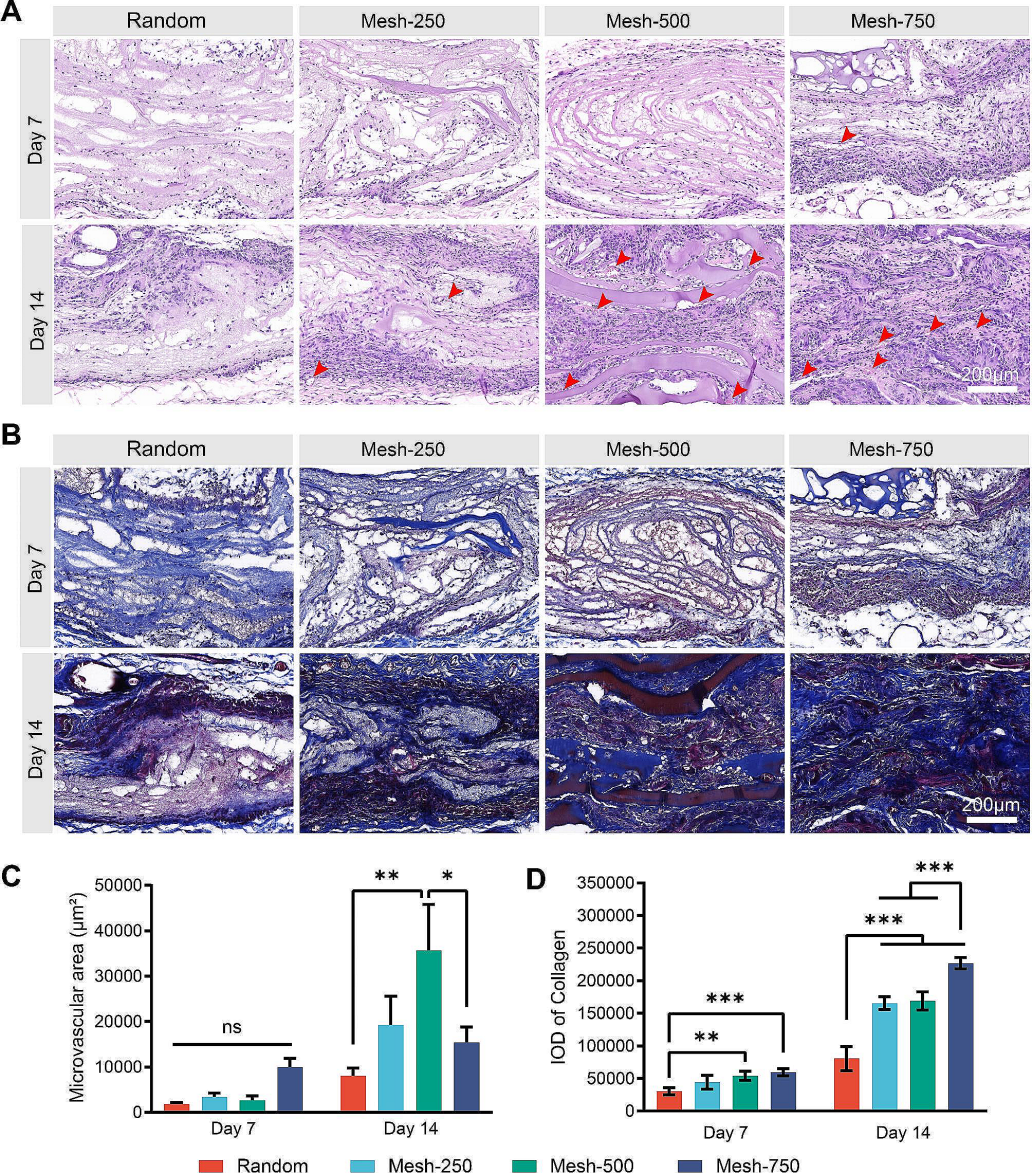
Tissue integration status of the scaffold in rat subcutaneous implantation. **(A)** HE staining of tissue sections. (red arrow: new vessels) **(B)** Masson’s staining of tissue sections. **(C)** Statistical analysis of vascular area within the scaffold. **(D) **Quantification of collagen deposition within the scaffold. (Data are represented as mean ± standard deviation, *n* = 5, ns, no significance, *P < 0.05, **P < 0.01, ***P < 0.001)

3D scaffolds were implanted subcutaneously in rat dorsa to observe tissue integration speed and potential immune reactions. In sections stained with Hematoxylin and Eosin (HE), the cytoplasm appeared red while nuclei were stained purple. Non-specific staining was observed on undegraded material: the electrospun membrane was pink and the hydrogel presented a light purple hue (Fig. 5A). On Day 7, a greater number of cells were found surrounding the scaffold than within it, and the infiltration areas expanded with the increment in mesh size. On Day 14, both Mesh-500 and Mesh-750 scaffolds were densely populated by cells, save for some remaining undegraded hydrogel. Conversely, the Random scaffold and Mesh-250 exhibited regions without any cells. The microvascular area was measured, there was no statistical significance on day 7 across samples. However, by the second week, the Mesh-500 scaffold displayed the highest vascularization, registering significant differences when compared to the Random and Mesh-250 scaffolds (Fig. 5C). Using Masson’s trichrome stain, collagen deposits were detected within the material. Collagen deposition was faint on day 7, evidenced by the predominance of light blue regions. On day 14, this deposition surged notably, both Mesh-500 and Mesh-750 showed intense blue collagen areas. In contrast, the Random scaffold demonstrated minimal collagen presence, closely followed by Mesh-250 (Fig. 5B). The Integral optical density (IOD) of blue collagen was measured. On day 7, the Random scaffold had the least IOD. On day 14, Mesh-750 showed the largest IOD, followed by Mesh-500 and Mesh-250 (Fig. 5D). This data indicates that scaffolds with larger mesh pores are more conducive to rapid cell infiltration and collagen deposition, yet Mesh-500 offers the most substantial microvascular area.

### Immune response of the scaffold in rat subcutaneous implantation

**Fig. 6:**
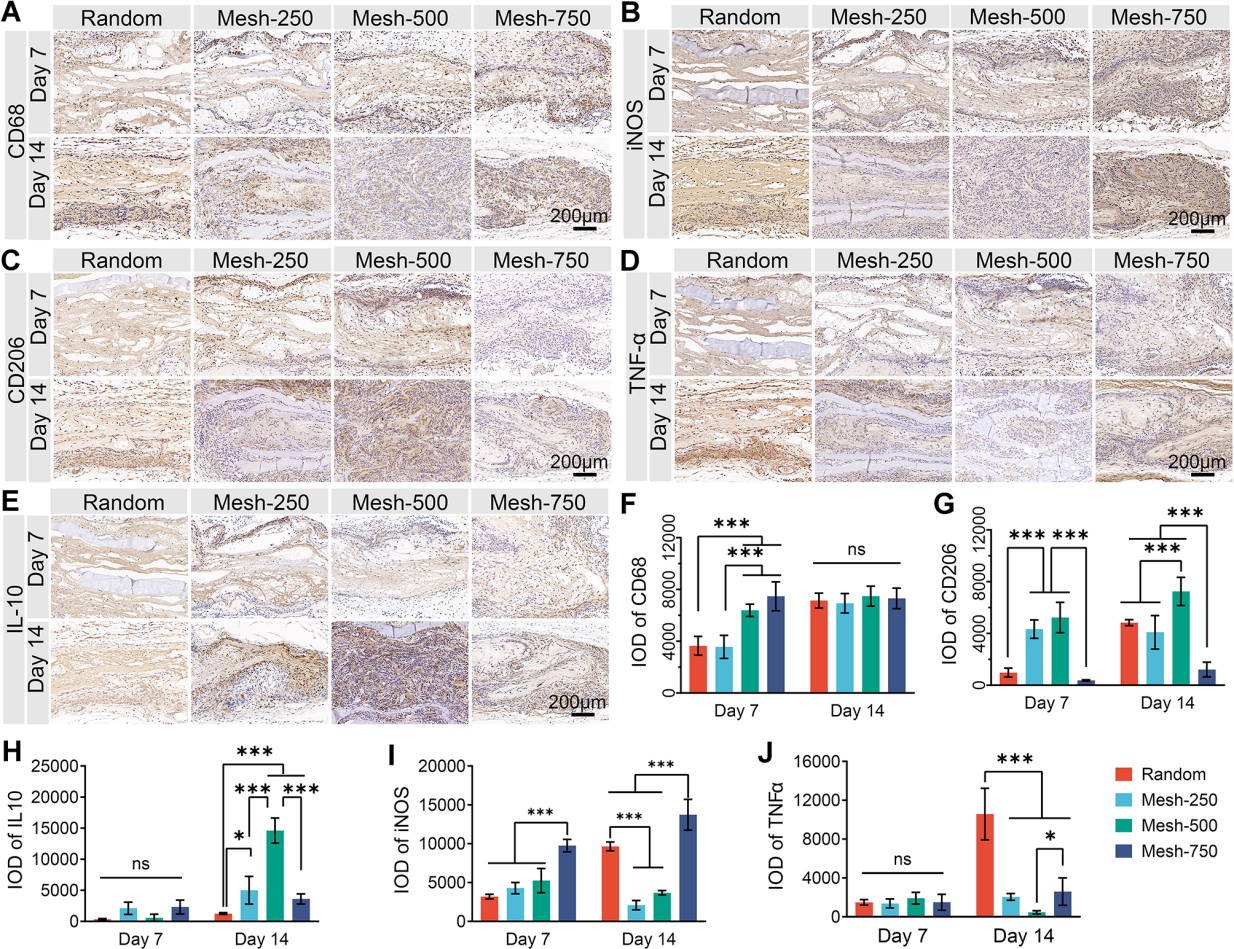
Immune response of the scaffold in rat subcutaneous implantation. **(A-E)** Immunohistochemistry staining evidencing the presence of CD68, iNOS, CD206, TNF-α, and IL-10. **(F-J)** Integrated optical density (IOD) measurements for CD68, CD206, IL-10, iNOS, and TNF-α. (Data are represented as mean ± standard deviation, *n* = 5, ns, no significance, *P < 0.05, **P < 0.01, ***P < 0.001.)

To assess the immunogenic responses triggered by scaffolds of diverse structures when placed subcutaneously, immunohistochemical analyses were performed for macrophage polarization markers and their correlating cytokines. CD68 is a pan-macrophage marker (Fig. 6A). iNOS and TNF-α typify the M1 macrophage phenotype (Fig. 6B, D), whereas CD206 and IL-10 are representative of the M2 macrophage phenotype (Fig. 6C, E). On Day 7, Mesh-500 and Mesh-750 displayed elevated IOD values for CD68 compared to Random and Mesh-250, presumably due to enhanced cell infiltration through the expansive pores of these scaffolds (Fig. 6F). A divergence in CD206 and iNOS levels indicated that by Day 7, Mesh-500 presented a higher concentration of M2 cells and fewer M1 cells than Mesh-750 (Fig. 6G, I). On Day 14, there was an absence of significant variation in the CD68 values across the scaffolds, suggesting substantial macrophage migration (Fig. 6A). CD206 demonstrated elevated expression in the Random, Mesh-250, and Mesh-500 scaffolds (Fig. 6C, G), with IL-10 peaking in Mesh-500 (Fig. 6E, H). Both the Random and Mesh-750 scaffolds revealed heightened iNOS expression, contrasted by Mesh-250 and Mesh-500 (Fig. 6B, I). TNF-α exhibited a pronounced expression solely in the Random scaffold (Fig. 6D, J). In summary, while Mesh-500 and Mesh-750 promoted swift immunocyte migration into scaffolds, Mesh-500 appeared to favor the M2 macrophage polarization. The Random scaffold, however, supported slower cell infiltration paired with a significant expression of M1 macrophage indicators.

Subcutaneous implantation of materials has been leveraged to investigate tissue ingrowth into materials, the ensuing immune responses, material degradation, and neo-tissue formation [30, 31]. Post-implantation, materials undergo sequential phases including injury, hemostasis-material interaction, provisional matrix formation, acute inflammation, chronic inflammation, granulation tissue development, and fibrous capsule development [32, 33]. In brief, during protein adsorption and acute inflammation, as a biomaterial is integrated into the host, proteins from the bloodstream swiftly adsorb onto the material’s surface, recruiting leukocytes like neutrophils and macrophages, and instigating an acute inflammatory reaction. If macrophages fail to phagocytize and degrade the biomaterial, they might fuse, giving rise to multinucleated foreign body giant cells. These cells persistently attempt to disintegrate the material, releasing various cytokines and enzymes. Fibrous encapsulation emerges during prolonged inflammation and foreign body reactions, where fibroblasts and smooth muscle cells are recruited, synthesizing and secreting collagen, thereby forming a dense fibrous capsule, and isolating the biomaterial from adjacent healthy tissue [34, 35]. Notably, no prominent fibrous capsules were discerned around these four scaffold scaffolds. This could be attributed to the nanofibrous scaffolds minimizing inflammatory responses, coupled with the porous structure fostering cellular infiltration [36]. Moreover, the thin electrospun membrane enables macrophages and foreign body giant cells to effectively encapsulate the spun layer, reducing the release of indigestible foreign signals.

Macrophages, intrinsic immune cells, play pivotal roles in foreign body reactions and wound healing following biomaterial implantation. In foreign body reactions, macrophages can polarize into distinct phenotypes, primarily M1 (pro-inflammatory) and M2 (anti-inflammatory) [37]. These two macrophage phenotypes distinctly influence tissue repair. M1 macrophages are predominantly active during initial injury and inflammatory responses, releasing pro-inflammatory cytokines like Tumor Necrosis Factor-alpha (TNF-α) and Interleukin-1β (IL-1β), along with antimicrobial and destructive oxidants and enzymes. While M1 macrophages mitigate pathogens and dead cells in the wounded area through inflammation, an excessive M1 response might lead to exacerbated inflammation and tissue damage. Conversely, M2 macrophages, primarily active during the latter stages of wound healing, emit anti-inflammatory cytokines such as Interleukin-10 (IL-10) and Transforming Growth Factor-beta (TGF-β), and factors that boost tissue repair and remodeling. By dampening excessive inflammation, M2 macrophages promote cellular proliferation, matrix synthesis, and subsequently, tissue regeneration and repair. However, an overdriven M2 response might culminate in excessive matrix deposition and fibrosis [38, 39]. Prior research intimates that mesh-like membranes, in comparison to random and aligned topography membranes, were advantageous to immunocyte recruitment and angiogenesis. Moreover, in the bone microenvironment, they favored the upregulation of M2 macrophage marker gene expression [15]. This aligns with our empirical findings, wherein Mesh-500 notably fostered macrophage polarization towards the M2 phenotype. This phenomenon may be linked to the orderly alignment of nanofibers on the Mesh-500 membrane and the membrane’s porosity. Furthermore, overlaying multiple membranes intensifies the topographical differences instigated by the membrane. Previous studies have reported that perforated CO_2_ expanded nanofibrous scaffolds favored an increased M2/M1 ratio four weeks post-implantation, highlighting the importance of the porosity of nanofibrous scaffolds [40]. In contrast with Mesh-500, while the sparser threads in the Mesh-750 scaffold facilitated faster cellular penetration to the scaffold’s center, M2 macrophage marker expression was not as elevated as in Mesh-500. This suggests that rapid cellular infiltration isn’t necessarily optimal. However, this might also be related to the thicker edges of the Mesh-750 grid, compelling infiltrating macrophages to expend more time degrading these edges. Earlier studies indicated that angiogenesis and scaffold vascularization necessitate the coordinated efforts of both M1 and M2 macrophages [41, 42]. The augmented vascular area in the Mesh-500 scaffold might result from its elicited harmonized immune responses.

### Mesh-500 scaffold modulates macrophage polarization by activation of PI3K/AKT signaling pathway in vivo

**Fig. 7:**
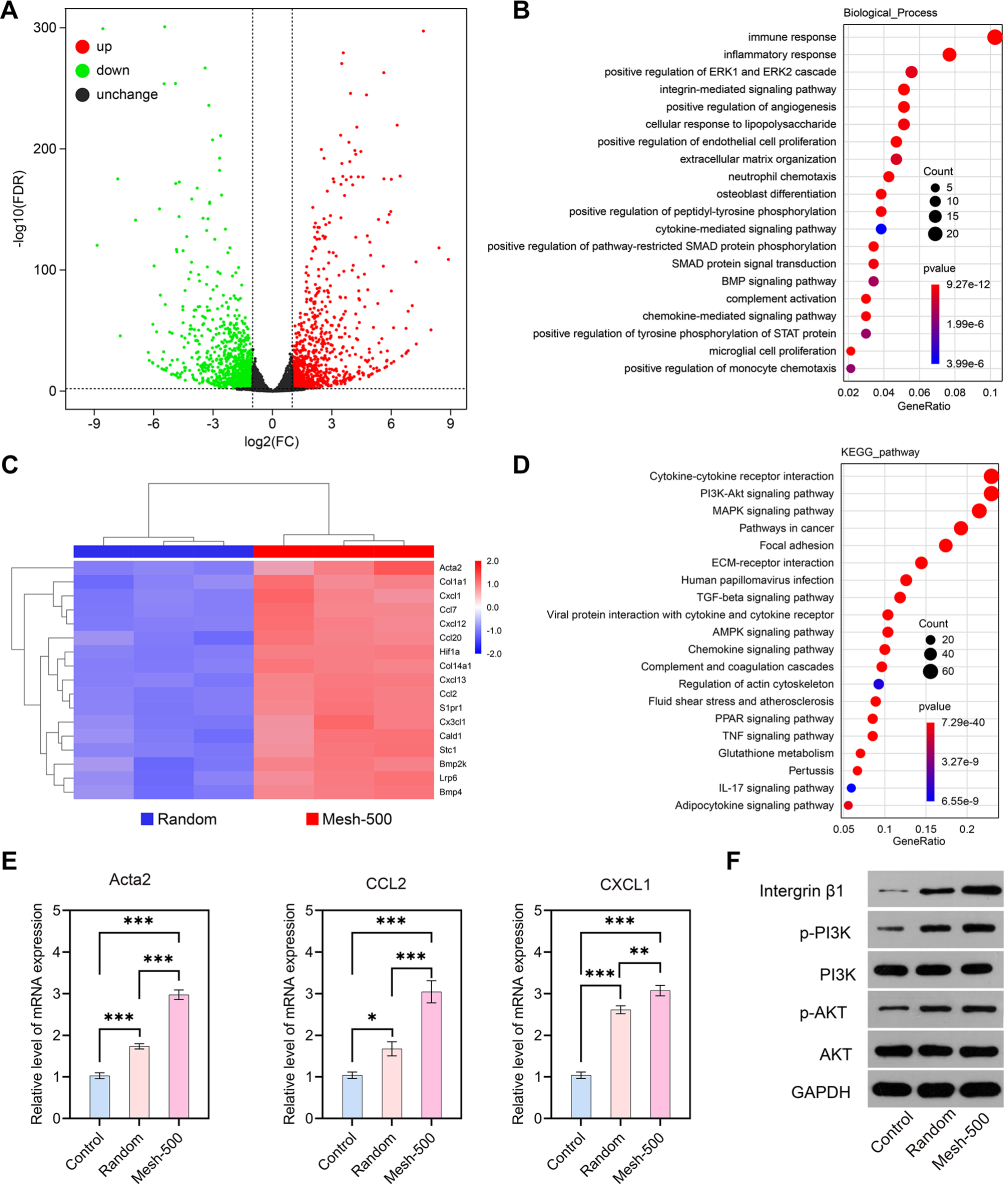
RNA-seq, GO/KEGG analysis, and verification of the PI3K/AKT pathway. **(A)** The volcano plot of differential expression of genes between Random and Mesh-500 group: the up-regulated, unchanged genes, and down-regulated were doted in red, black, and green, respectively. **(B)** GO enrichment analysis for differential expressed genes (biological process). (**C)** A cluster heatmap showed differentially expressed genes involved in PI3K/AKT signaling pathway (|Log2 FC| > 1). **(D)** The top twenty enriched signaling pathways following KEGG enrichment analysis for differential expressed genes. **(E)** Relative mRNA levels of genes, including Acta2, CCL2, and CXCL1, as determined by qRT-PCR. **(F)** Representative Western blot images of RAW264.7 cells cultured on the Random and Mesh-500 group. (*n* = 3). (Data are represented as mean ± standard deviation, n = 3, ns, no significance, *P < 0.05, **P <0.01, ***P < 0.001.)

We conducted RNA-seq analysis on both the Random scaffold and Mesh-500 scaffold two weeks post-subcutaneous removal to decipher the underlying mechanisms accounting for their divergent outcomes. Differential gene expression analysis revealed that in the Mesh-500 scaffold, 1031 genes were upregulated and 1261 genes were downregulated with a significance level of *p* < 0.05 and an absolute log2 fold change greater than 1 (Fig. 7A). Notably, the Mesh-500 scaffold augmented the expression of pivotal immune-related genes, including Acta2, CCL2, and CXCL1 (Fig. 7C, E). Gene Ontology (GO) analysis highlighted a pronounced enrichment of immune-associated genes, primarily clustered into categories such as “immune response,” “inflammatory response,” “extracellular matrix organization,” and “positive regulation of monocyte chemotaxis.” (Fig. 7B) Subsequent KEGG pathway analysis identified twenty significant associated targets and pathways. Notably, the PI3K/AKT signaling pathway, a known regulator of M2 polarization, saw a marked increase (Fig. 7D). It’s established that a material’s topological morphology influences the adherence of macrophages on its surface. This adherence subsequently fosters macrophage differentiation, primarily driven by integrin expression. Phosphatidylinositol 3 kinase (PI3K), a precursor regulator of protein kinase B (AKT), modulates the M2 macrophage phenotype [43]. Activation of PI3K catalyzes the conversion of phosphatidylinositol 4,5-bisphosphate (PIP2) to phosphatidylinositol 3,4,5-trisphosphate (PIP3). This latter molecule is pivotal for Akt activation as it binds to the PH domain, phosphorylating and activating Akt, thereby amplifying M2-associated gene expression [44]. Further assessment via the Western Blot assay of macrophage integrin/PI3K/AKT pathway-related proteins on the scaffolds revealed a progressive increase in the relative expression of integrin, p-PI3K/PI3K, and p-AKT/AKT across the Control, Random, and Mesh-500 scaffolds (Fig. 7F, Fig. S4). This pattern suggests that the Mesh-500 scaffold more effectively activates the PI3K/AKT pathway within macrophages.

### Mesh-500 scaffold modulates macrophage polarization by activation of PI3K/AKT signaling pathway in vitro

**Fig. 8:**
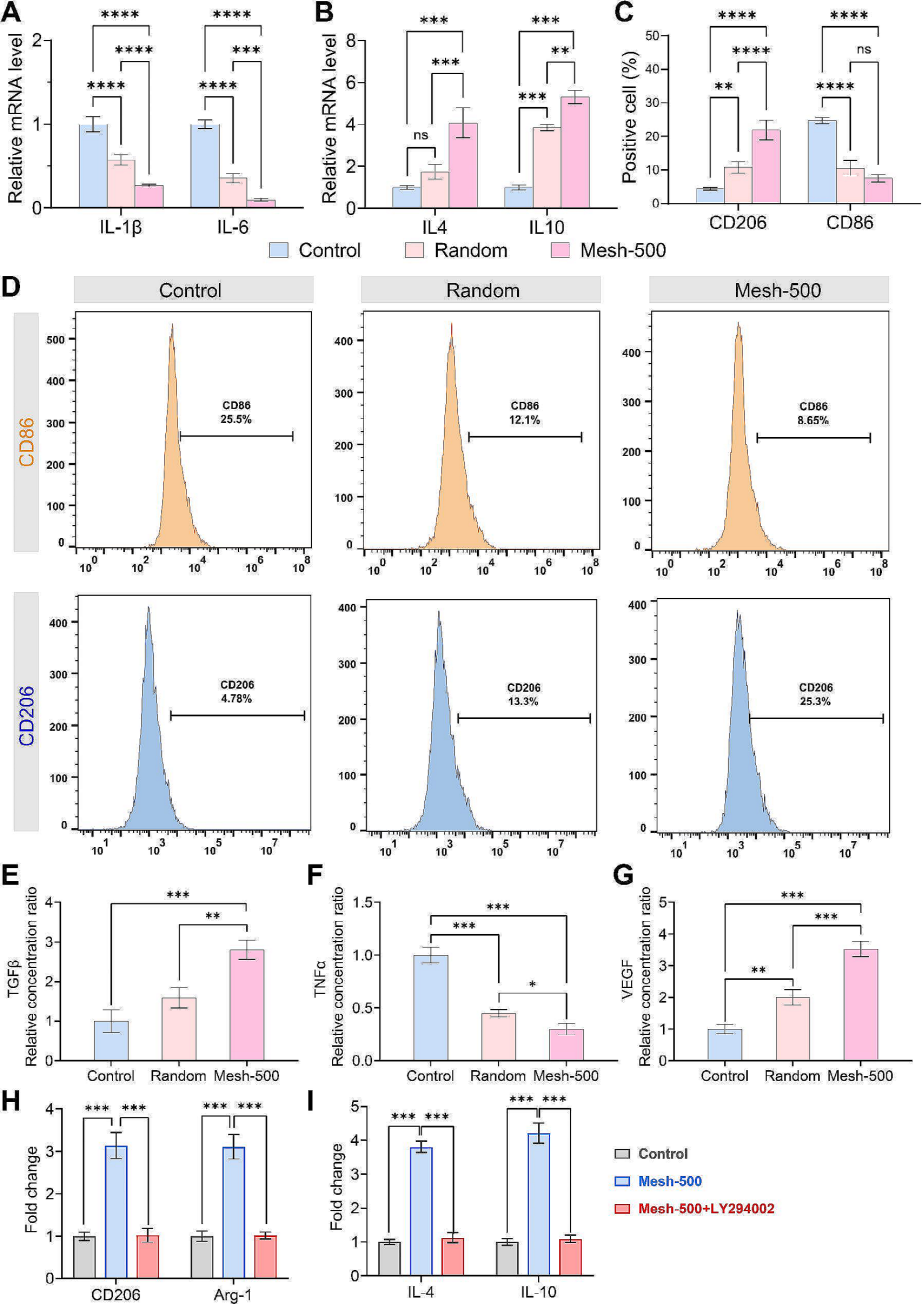
The phenotype of the macrophages cultured on the Random and Mesh-500 group. **(A, B)** Relative mRNA levels of genes, including IL-1β, IL-6, IL-4, and IL-10, as determined by qRT-PCR. The data were normalized to that of the control cells seeded on tissue cell plates. **(C)** Quantitative results of FCA. **(D)** Expression of CD206 and CD86 in RAW264.7 cells by FCA. **(E-G)** The concentrations of TGF-β, TNF-α, and VEGF in the cell supernatant were determined by ELISA. **(H, I)** The fold change of mRNA levels of genes, including CD206, Arg-1, IL-4, and IL-10. (Data are represented as mean ± standard deviation, *n* = 3, ns, no significance, *P < 0.05, **P < 0.01, ***P < 0.001.)

We subsequently sought to discern the influence of the scaffold on macrophages, following a seven-day in vitro intervention. PCR analyses revealed that the expression of M1 macrophage-specific markers, IL-1β and IL-6 mRNA, exhibited a decline across the Control, Random, and Mesh-500 cohorts (Fig. 8A). Conversely, M2 macrophage indicators, namely IL-4 and IL-10, demonstrated an ascending trajectory in the aforementioned groups (Fig. 8B). Flow cytometric analysis further corroborated that the Mesh-500 cohort was more conducive to the augmentation of CD206 markers, while simultaneously witnessing a decline in CD86 markers on the macrophage surface, in comparison to the Control and Random group (Fig. 8C, D). Sequentially, we evaluated the secretion levels of TGFβ, TNFα, and VEGF in the supernatant via the ELISA methodology (Fig. 8E, F, G). It was discerned that while TGFβ and VEGF were substantially augmented in the Mesh-500 group, TNFα was markedly diminished. Introducing the PI3K inhibitor, LY294002, to the Mesh-500 cohort for macrophage treatment led to intriguing observations. Seven days post-treatment, the mRNA expression levels of CD206 and Arg-1 in macrophages mirrored those of the Control group (Fig. 8H). Concurrently, the excretion of IL-4 and IL-10 in the supernatant realigned to concentrations analogous to the Control group (Fig. 8I). Cumulatively, these observations provide compelling evidence that the Mesh-500 scaffolds modulate macrophage polarization towards an M2 phenotype via the PI3K/AKT signaling pathway in vitro.

This study acknowledges certain limitations. Specifically, the hydrogel’s geometrical attributes, which function as a foundational pillar in the electrospun membrane layer, significantly influence the scaffold’s structure. Factors such as pattern shape, pattern density, and gel layer height were not rigorously examined in this research. Furthermore, extending the observation period for the subcutaneous implantation model might have offered insights into tissue attributes post-complete material degradation.

## Conclusions

In conclusion, we have successfully demonstrated the feasibility of 3D printing hydrogels as pillars on electrospun membranes to enable the assembly of membrane layers to obtain three-dimensional scaffolds. We fabricated four kinds of porous 3D nanofiber scaffolds with random membranes and three kinds of mesh-like membranes with different mesh sizes. The nanofibers are composed of PCL and silk fibroin, the addition of silk can improve the hydrophilicity and biocompatibility of PCL, and modulate the negative immune response. These scaffolds have good mechanical properties to meet the needs of tissue engineering. In the in vitro cell inoculation experiments, the mesh-like membrane was more favorable to cell proliferation than the random electrospinning membrane, and the larger the mesh size was more favorable to cell penetration through the electrospinning membrane. During subcutaneous trials, the larger the mesh pores were more favorable for rapid inward cell penetration. Nevertheless, our findings suggest that an optimal mesh pore dimension, specifically Mesh-500, optimally directs macrophage polarization within the scaffold toward the M2 phenotype. This phenomenon potentially fosters a conducive microenvironment for tissue restoration, expediting tissue growth, matrix deposition, and scaffold vascularization. Consequent RNA sequencing tests and wet lab experiments inferred that the Mesh-500’s distinct topology might modulate macrophage polarization towards the M2 phenotype via the PI3K/AKT pathway’s interaction. Recognizing the unique microenvironments of various implantation sites (e.g., bone, skin, cartilage, muscle), future research will delve into the 3D scaffold’s applicability as a tissue-engineering platform.

### Electronic supplementary material

Below is the link to the electronic supplementary material.

Supplementary Material 1

Supplementary Material 2

## Data Availability

The raw data related to this work are available from the corresponding author upon reasonable request.
