# Supplementary material for: 3D nanofiber scaffolds from 2D electrospun membranes boost cell penetration and positive host response for regenerative medicine
Source: J Nanobiotechnology. 2024 Jun 8;22:322. doi: 10.1186/s12951-024-02578-2 (PMC11162076; doi:10.1186/s12951-024-02578-2)
Supplement: Supplementary file 2 — Supplementary Material 2 [file 12951_2024_2578_MOESM2_ESM.docx]

**Supporting information**

**3D Nanofiber Scaffolds from 2D Electrospun Membranes Boost Cell Penetration and Positive Host Response for Regenerative Medicine**

Lingfei Xiao ^a, †^, Huifan Liu ^b, †^, Huayi Huang ^a, †^, Shujuan Wu ^c^, Longjian Xue ^d^, Zhen Geng ^e, f *^, Lin Cai ^a, *^, Feifei Yan ^a, *^

*a Department of Spine Surgery and Musculoskeletal Tumor, Zhongnan Hospital of Wuhan University, 168 Donghu Street, Wuchang District, Wuhan 430071 Hubei, China.*

*b Department of Anesthesiology, Research Centre of Anesthesiology and Critical Care Medicine, Zhongnan Hospital of Wuhan University, 168 Donghu Street, Wuchang District, Wuhan 430071 Hubei, China.*

*c Department of Respiratory and Critical Care Medicine, Renmin Hospital of Wuhan University, Wuhan, China.*

*d The Institute of Technological Science, School of Power and Mechanical Engineering, Wuhan University, 430072 Wuhan, China.*

*e Institute of Translational Medicine, Shanghai University, Shanghai, 200444, China*

*f National Center for Translational Medicine (Shanghai) SHU Branch, Shanghai University, Shanghai, 200444, China*

^†^ These authors contribute equally to this work

* Corresponding authors: orthopedics@whu.edu.cn (Lin Cai), yanfeifei0120@whu.edu.cn (Feifei Yan), nanboshan1987@163.com (Zhen Geng)

**1. Supplementary figures**


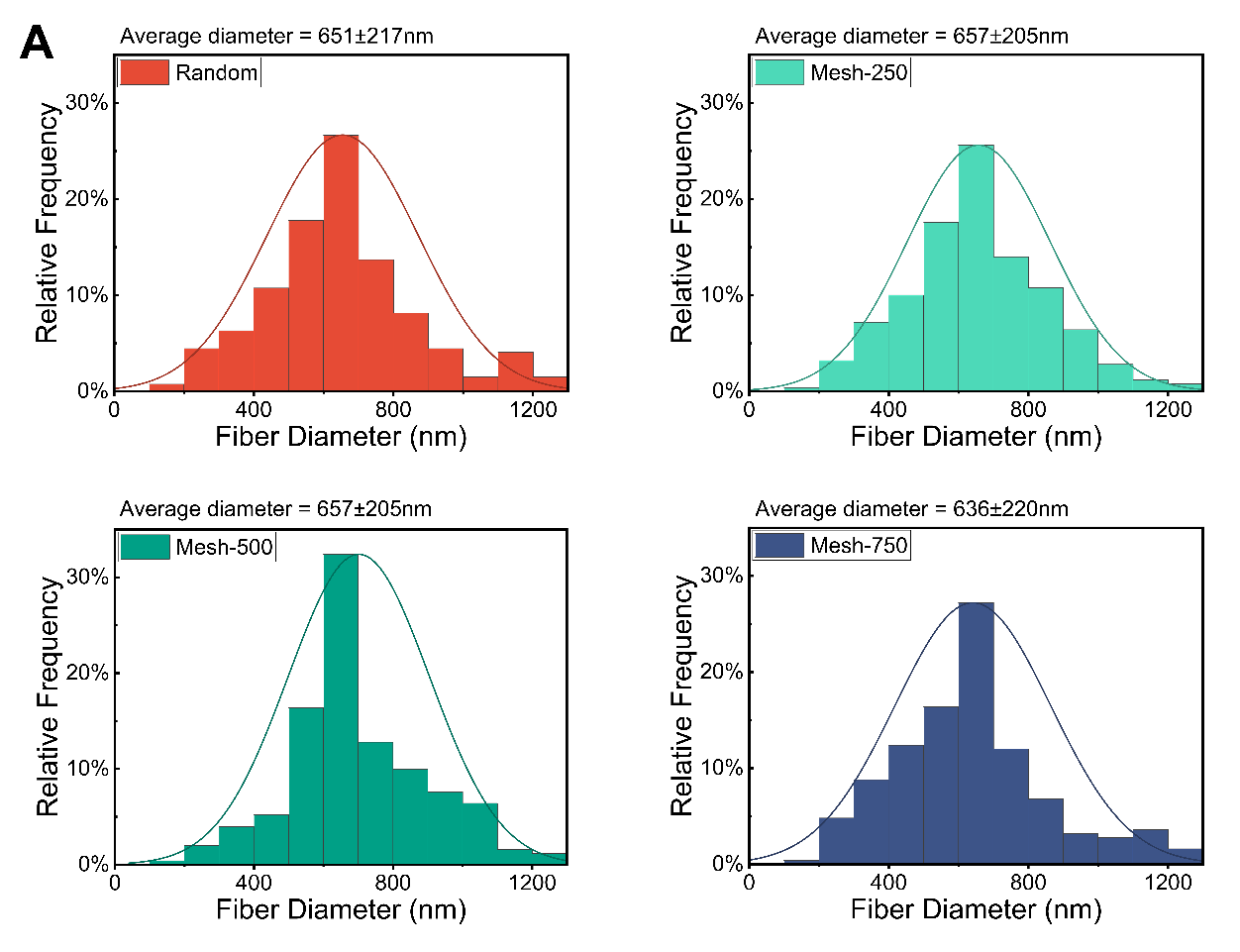


**Figure S1.** (A) Diameter distribution of the electrospun nanofiber membranes.


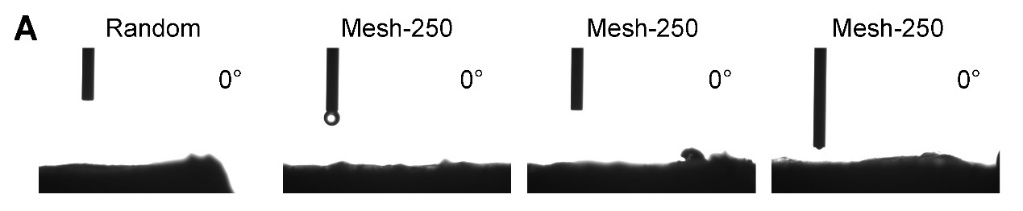


**Figure S2.** (A)  Images of the water contact angle of Random, Mesh-250，Mesh-500，Mesh-750.


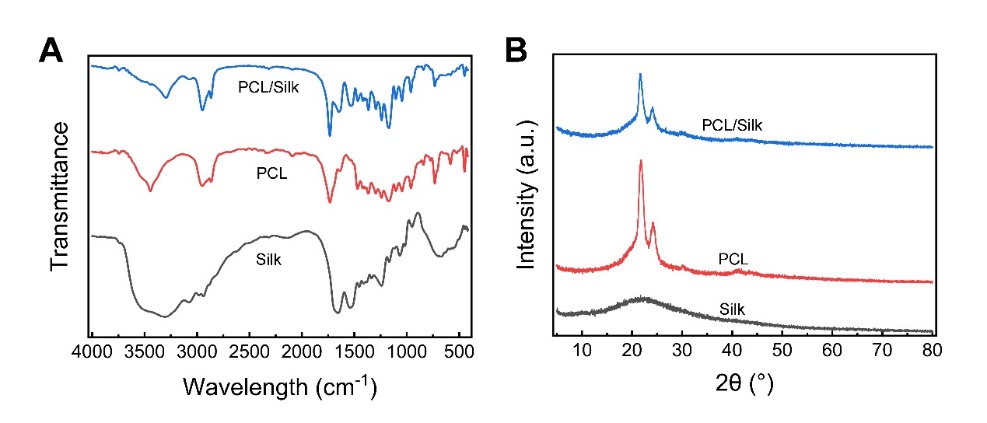


**Figure S3.** (A) FTIR spectra of Silk, PCL and PCL/ Silk. (B) XRD patterns of Silk, PCL and PCL/ Silk.


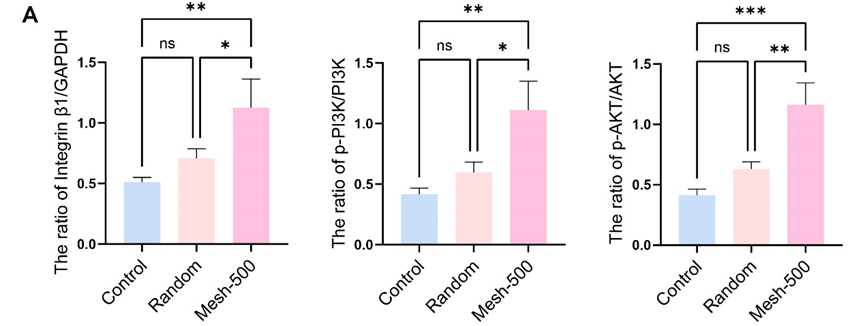


**Figure S4.** (A)  Quantification of the target proteins. ns, no significance, *P < 0.05, **P < 0.01, ***P < 0.001.

**2. Supplementary tables**

**Table S1**. Parameters of the prepared 3D nanofibrous scaffolds

|  | Volume(mm3) | Weight(g) | Weight in water for 24 hours)(g) | Porosity (%) |
| --- | --- | --- | --- | --- |
| Random | 125 | 0.029±0.008 | 0.123±0.049 | 75.3±8.4 |
| Mesh-250 | 125 | 0.030±0.011 | 0.119±0.031 | 79.5±11.5 |
| Mesh-500 | 125 | 0.027±0.006 | 0.107±0.027 | 81.3±9.9 |
| Mesh-750 | 125 | 0.031±0.015 | 0.145±0.034 | 82.8±12.1 |

| **Table S2.** Primer sequences used in RT-qPCR analysis. | |
| --- | --- |
| Genes | Primers sequences |
| Mouse-GAPDH-F | TCAACGGCACAGTCAAGG |
| Mouse-GAPDH-R | TTAGTGGGGTCTCGCTCC |
| Mouse-IL-1β-F | TGTGTTTTCCTCCTTGCCTCTGAT |
| Mouse-IL-1β-R | TGCTGCCTAATGTCCCCTTGAAT |
| Mouse-IL6-F | GAGACCACTGGGGAGAATGC |
| Mouse-IL6-R | TTGCCAGGTGGGTAAAGTGG |
| Mouse-IL-4-F | GGTCTCAACCCCCAGCTAGT |
| Mouse-IL-4-R | GCCGATGATCTCTCTCAAGTGAT |
| Mouse-IL10-F | TTTCAAACAAAGGACCAG |
| Mouse-IL10-R | GGATCATTTCCGATAAGG |
| Mouse-CD206-F | GCAAGTGATTTGGAGGCT |
| Mouse-CD206-R | ATAGGAAACGGGAGAACC |
| Mouse-Arg-1-F | AAGACAGCAGAGGAGGTG |
| Mouse-Arg-1-R | AGTCAGTCCCTGGCTTAT |
| Rat-GAPDH-F | CTCCCATTCTTCCACCTTTG |
| Rat-GAPDH-R | TGGTCCAGGGTTTCTTACT |
| Rat-Acta2-F | TGAACCCTAAGGCCAACCG |
| Rat-Acta2-R | TCCAGAGTCCAGCACAATACCA |
| Rat-CCL2-F | CCCAGAAACCAGCCAACT |
| Rat-CCL2-R | TGCTGCTGGTGATTCTCTTG |
| Rat-CXL1-F | GCAGACAGTGGCAGGGATT |
| Rat-CXL1-R | GGACACCCTTTAGCATCTTTT |
